# Mental Health of Flying Cabin Crews: Depression, Anxiety, and Stress Before and During the COVID-19 Pandemic

**DOI:** 10.3389/fpsyg.2020.581496

**Published:** 2020-12-17

**Authors:** Yvonne Görlich, Daniel Stadelmann

**Affiliations:** Department of Psychology, PFH Private University of Applied Sciences, Göttingen, Germany

**Keywords:** depression, anxiety, stress, working conditions, unemployment, aerospace, aviation, COVID-19

## Abstract

**Objectives:** Initially, we analyzed relations between the challenging working conditions of flight attendants with symptoms of depression, anxiety and stress. As the COVID-19 pandemic plunged airlines into an unprecedented crisis, its impact on the mental health of flying cabin crews became the focus of a second survey.

**Methods:** Flight attendants were surveyed online with DASS-21 in May 2019 (*N* = 105; sample 1) and April 2020 (*N* = 1119; sample 2), complemented with questions about working conditions (in 2019) and existential fears and fear of job loss (in 2020).

**Results:** Sample 1 revealed that symptoms of depression, anxiety and stress highly correlated with the subjective assessment of working conditions, but not with objectifiable parameters. Sample 2 showed significant positive correlations between existential fears and fear of job loss with depression, anxiety and stress. Crew members, grounded in April 2020, showed significantly higher scores in depression and stress, while still flying individuals had more clinically relevant symptoms of anxiety. Mean value comparisons between sample 1 and 2 in DASS-21 revealed a significant increase in symptoms at the time of crisis with effect sizes of *d* = 0.63 for depression, *d* = 0.26 for anxiety, and *d* = 0.52 for stress. The incidence of clinically relevant symptoms among the respondents increased from 8 to 23% (depression), from 6 to 14% (anxiety), and from 8 to 24% (stress).

**Conclusion:** The COVID-19 pandemic and associated work restrictions coincide with severe impairment of mental health of flying cabin crews, consistent with a mental health protecting function of labor.

## Introduction

During their work, flight attendants are exposed to special health-related challenges. For example, McNeely and colleagues investigated the health status of cabin crews and found significantly more sleep problems, depression, anxiety, and fatigue than in the average population (McNeely et al., 2014, 2018). Fatigue is favored by night work, a very early start of work, long flying hours, long time shifts, and impairment of the biorhythm. In the case of national flights, a very early beginning or very late end of duty, but also irregularly structured duty schedules have been identified as problematic (Ono et al., 1991). Cabin crews on international flights suffer less from stress and fatigue than their national colleagues (MacDonald et al., 2003; Nagda and Koontz, 2003). In general, irregular working hours, due to shift work and/or night work, can increase the incidence of physical and psychological problems (Barton, 1994). In addition to objective factors, subjective stressors are also relevant (Frese and Zapf, 1988). Cabin crews are particularly affected by anxiety and post-traumatic stress following air accidents (Dyregrov et al., 1992; Marks et al., 1995). Although the vast majority of flights run without incident, up to 37% of cabin personnel feel anxiety before take-off. According to Suvanto and Ilmarinen (1989), both cognitive and physical overloads are the beginning of flight-related work stress. A Norwegian survey showed that only half of the flying cabin crew members were satisfied with their social support (Skogstad et al., 1995). Work-related emotionality is reflected in specific requirements, for example, in the cultural and linguistic differences between cabin crews and passengers, with emotional exhaustion increasing with age (Chang and Ju-Mei, 2009) and with emotional dissonance (Zapf and Holz, 2006).

In the present study, a sample of May 2019 examined the connection between various condition factors such as fatigue or workload and symptoms of depression, anxiety, and stress. As conditions for flight attendants changed dramatically with the COVID-19 pandemic, an additional survey was conducted in April 2020 in order to investigate the impact of the crisis on aspects of mental health and especially changes in the symptoms of depression, anxiety and stress.

The COVID-19 pandemic has been spreading from China across the world since the beginning of 2020. In Europe, the first infection wave peaked in April. Following a relaxation during summer, a second wave with even higher infection numbers hit the continent with the cold season’s onset in late October. According to WHO data from November 15, 2020, over 53 million people worldwide were confirmed to be infected, and over 1,300,000 people have died (WHO, 2020). In order to contain the virus, many countries have implemented curfews, contact bans, closed restaurants, schools, kindergartens, nurseries, borders, and travel restrictions.

The financial crisis (2007–2009) led to a rise in unemployment, increased workloads, staff cuts, and wage cuts, resulting in an increase in the incidence of anxiety disorders, depression, dysthymia, and suicide (Mucci et al., 2016). The corona crisis was also associated with an increase in depression, anxiety, and stress symptoms; this has been studied, e.g., in China (Huang and Zhao, 2020; Lai et al., 2020; Wang et al., 2020; Xing et al., 2020), Mongolia (Otgonbaatar et al., 2020), Spain (Ozamiz-Etxebarria et al., 2020), Italy (Cerami et al., 2020) and Switzerland (de Quervain et al., 2020). These more recent studies looked at the general population or healthcare workers in hospitals. They did, however, not specifically investigate the crisis’ impact on employees of the most crisis-affected industries. Mental health challenges faced by India’s internal migrant workers as a result of the COVID 19 pandemic were highlighted (Choudhari, 2020).

Indeed, the economic impacts caused by anti-pandemic containment measures vary widely between industries. Online trade or other digital industries have benefited greatly. On the other hand, the airline industry is severely affected by the restrictions, not only because of the formal travel bans but also because of voluntary restrictions, such as replacing business trips with online meetings. The largest German airline Lufthansa, for example, lost 1 million euros per hour in April according to its own figures; and passenger numbers have fallen by 99% from daily 350,000 to 3,000 (Spohr, 2020).

The financial consequences for these companies are indeed dramatic; many airlines are already insolvent. In contrast to the situation in other countries, however, the larger companies in Germany have (so far) refrained from dismissing personnel. Instead, they are resorting to instruments such as “Kurzarbeit” (short-time work), in which the Federal Employment Agency (unemployment insurance) temporarily compensates the employees and thereby relieves the companies financially. Lufthansa, but also other airlines, have announced short-time work for 5 months. Short-time work is a reduction in working hours (in extreme cases down to zero), with the resulting loss of pay being partially compensated by a state insurance scheme (to which employees and employers have previously paid contributions). Lufthansa justified this step with an enormous reduction in its active fleet: by April, 700 of 760 aircraft were not in use (Sueddeutsche.de, 2020). However, the short-time work allowance is significantly lower than the regular net remuneration; and since this is a temporary measure, later layoffs cannot be ruled out.

How are flight attendants, the largest employee group in the air traffic industry, reacting to this crisis? One assumption could be that the situation is viewed positively, because negatively perceived working conditions and consequences such as fatigue, jet lag, and physical and mental workloads are alleviated. At the same time, they have plenty of free time and can return to more balanced sleeping habits. However, this is offset by the burden of inadequate childcare (with schools, kindergartens, and nurseries being closed), significant financial losses, the possible fear of contracting SARS CoV-2 along with a severe course of the disease, worries about relatives and friends, as well as uncertainty about the future of their employment.

## Materials and Methods

### Study Design

Samples were acquired through a web-based online survey (LimeSurvey). Participants were recruited through the network of the co-author DS (himself being a flight attendant) and by placing links to the survey on websites that are frequently visited by flight attendants, namely the websites of the independent Union of Flight Attendants (UFO)^1^ and of aero.de.^2^ The surveys were conducted in German. Sample 1 (May 2019) covered the survey period 14, May 2019 to 23, May 2019 and sample 2 (April 2020) the period from 8, April 2020 to 28, April 2020. The surveys were anonymous and voluntary after informed consent, and participants could stop the survey at any time. This study was performed in accordance with the Declaration of Helsinki and approved by the Ethics Committee of the PFH Göttingen.

### Samples

Hundred and five employees of 12 airlines participated in the survey in May 2019 (sample 1) and 1119 employees of 22 airlines in the survey in April 2020 (sample 2). The 10 most frequently named airlines account for 95% of participants of survey 1 and for 90% of participants of survey 2. More details of the sample description can be found in Table 1.

**Table 1:** Sample characteristics.

	Sample 1, May 2019		Sample 2, April 2020	
N	105		1119	
**Gender**				
Male	33 (31.4%)		201 (18.0%)	
Female	72 (68.6%)		912 (81.9%)	
Diverse	0 (0%)		1 (0.1%)	
**Position**				
Flight attendant	68 (64.8%)		963 (86.2%)	
Purser/ette	37 (35.2%)		154 (13.8%)	
**Employment contract**				
Fixed-term	3 (2.9%)		47 (4.2%)	
Permanent	102 (97.1%)		1071 (95.8%)	
Flight profile			Before corona	During corona
National and short haul (up to 2 h)	4 (3.8%)		29 (2.6%)	23 (2.1%)
Medium haul (up to 5 h)	11 (10.5%)		48 (4.3%)	1 (0.1%)
Long haul (from 5 h)	6 (5.7%)		82 (7.3%)	22 (2.0%)
Mixed (national, short, and medium haul)	32 (30.5%)		166 (14.8%)	16 (1.4%)
Mixed (national, short, medium, and long haul)	52 (49.5%)		794 (71.0%)	81 (7.2%)
Non-flying (short-time work)	0 (0%)		0 (0%)	976 (87.2%)
	***M***	***SD***	***M***	***SD***
Age	38.50	9.83	31.47	9.97
Number of children	0.55	0.91	0.27	0.71
Years of work experience	13.69	9.07	8.31	8.29
Employment level	85.89%	17.46%	84.67%	18.82%

### Measurements

The German version (Nilges and Essau, 2015) of DASS-21 Depression Anxiety Stress Scales (Antony et al., 1998) were used to assess symptoms. This instrument measures depression, anxiety, and stress experienced during the last week, with seven items each (answer format: 0 = did not apply to me at all, 1 = applied to me to a certain degree or sometimes, 2 = applied to me to a considerable extent or quite often, and 3 = applied very strongly to me or most of the time). Thus, each scale had a range of 0–21. Cut-offs for clinically relevant symptoms are 10 for depression, 6 for anxiety and 10 for stress. The internal consistencies [Cronbach’s alphas (*α*)] were given by (Nilges and Essau, 2015) as 0.88 for depression scale, 0.76 for anxiety, and 0.86 for stress. In our study, Cronbach’s α was (sample 1/sample 2): 0.92/0.89 for depression, 0.70/0.77 for anxiety, and 0.86/0.87 for stress.

In both samples, additional subjectively assessed condition parameters were collected. We therefore designed a questionnaire on subjective assessments and also on more objective parameters. In sample 1, these refer to work-related job characteristics and subjective stressors. In sample 2, we asked for crisis-related changes in personal life situations, existential fears, and fear of losing one’s job (for details, see legend to Table 2).

**Table 2:** Correlation between DASS-scores and subjective evaluations, sample 2 April 2020.

S. No.		*M*	*SD*	Depression	Anxiety	Stress
1.	Fear of job loss	4.27	1.59	0.406^**^	0.295^**^	0.360^**^
2.	Existential fears	4.00	1.66	0.432^**^	0.356^**^	0.409^**^
3.	Existential fears before corona	1.99	1.07	0.080^**^	0.109^**^	0.124^**^
4.	Change in the personal situation	2.99	1.03	−0.445^**^	−0.277^**^	−0.393^**^
5.	Change in personal situation after corona	3.77	1.39	−0.164^**^	−0.083^**^	−0.166^**^

N = 1119, item contents: (1) Are you afraid of losing your job because of the corona crisis? (2) Do you currently have existential fears? (3) Did you have existential fears before the corona crisis? (answer options of these three items: 1 = not at all, 2 = no, 3 = rather no, 4 = partly - partly, 5 = rather yes, 6 = yes, 7 = yes, absolute); (4) Has your personal situation changed due to the corona crisis? (answer options: 1 = yes, it is now much worse, 2 = yes, it is now worse, 3 = yes, it is now rather worse, 4 = no, no change, 5 = yes, it is now rather better, 6 = yes, it is now better, 7 = yes, it is now much better); and (5) What do you expect for your personal situation after the corona crisis? (answer options: 1 = it gets much worse, 2 = it gets worse, 3 = it gets rather worse, 4 = no change, 5 = it gets rather better, 6 = it gets better, 7 = it gets much better).

* *p* < 0.05

** *p* < 0.01

*** *p* < 0.001.

Most aspects of job characteristics and subjective stressors have been analyzed by one or two items. These are detailed in the legends to Tables 3 and 4. “Fatigue,” “on-call duty,” and “duty roster design” were measured by multiple items, namely.

**Table 3:** Correlations between conditional factors and DASS-scores, sample 1 May 2019.

S. No.		*M*^+^	*SD*^+^	Depression	Anxiety	Stress
**Negative evaluation direction**						
1.	Time pressure/work intensity	2.77	0.64	0.210^*^	0.245^*^	0.329^**^
2.	Overload	0.90	0.77	0.139	0.238^*^	0.280^**^
3.	Underload/monotony	1.89	0.89	0.362^**^	0.046	0.164
4.	Physical strains	2.82	1.00	0.129	0.199^*^	0.214^*^
5.	Fatigue	2.09	0.78	0.483^**^	0.360^**^	0.529^**^
6.	Flight profile (psychol. demands)	1.90	1.03	0.425^**^	0.309^**^	0.519^**^
7.	Flight profile (physical demands)	2.43	1.00	0.374^**^	0.305^**^	0.361^**^
8.	On-call duty	2.84	1.08	0.294^**^	0.321^**^	0.454^**^
9.	Communication with guests	1.11	0.80	0.201^*^	0.166	0.233^*^
**Positive evaluation direction**						
10.	Physical conditions	2.66	0.78	−0.357^**^	−0.417^**^	−0.391^**^
11.	Climatic conditions	2.75	0.84	−0.102	−0.133	−0.177
12.	Cultural interactions with guests	3.33	0.63	0.157	0.112	0.233^*^
13.	Duty roster design	2.32	0.70	−0.248^*^	−0.123	−0.358^**^
14.	Working climate	2.93	0.79	−0.348^**^	−0.296^**^	−0.434^**^
15.	Support from colleagues	2.99	0.81	−0.068	0.038	−0.197^*^
16.	Support from supervisor	2.06	1.15	−0.251^**^	−0.009	−0.230^*^
17.	Appreciation by supervisor	1.90	1.19	−0.176	−0.066	−0.236^*^
**Without evaluation direction**						
18.	Fast decision-making	2.57	0.72	−0.116	0.104	0.078
19.	Perfect appearance	3.38	0.75	0.072	0.167	0.172
20.	Friendliness/permanent smile	3.06	0.82	0.098	0.126	0.105
21.	Irregular breaks	3.52	0.77	0.027	0.000	0.083
22.	Break in the turnaround	1.42	0.92	−0.224^*^	−0.086	−0.273^**^
23.	Retreat opportunities on board	1.35	1.17	−0.018	−0.018	0.005
24.	Irregular eating habits	3.35	0.75	0.250^**^	0.168	0.231^*^

N = 105, ^+^answer options: 0 = never; 1 = rarely, 2 = occasionally, 3 = often, 4 = always; item contents: (1) (Two items): I work under constant time pressure/I have a high work intensity, *r* = 0.56. (2) I feel overwhelmed in my work activity. (3) (Two items): I feel underloaded in my work activity/My work processes are monotonous, *r* = 0.59. (4) (Two items): Lifting and carrying guests’ luggage is physically demanding/Lifting and carrying boxes is physically demanding, *r* = 0.60. (5) For items, see method section. (6) I find my flight profile overall nerve-wracking. (7) I find my flight profile overall physically demanding. (8) For items, see method section. (9) Communication problems (e.g., language problems, English not included) in dealing with guests are a problem for me. (10) I deal well with physical conditions (e.g., air conditioning, pressurized cabin, and aircraft noise). (11) I deal well with climate differences between the various destinations. (12) I deal confidently with cultural differences in dealing with guests. (13) For items, see method section. (14) I would describe the working atmosphere as pleasant. (15) I get personal support from my colleagues. (16) I receive personal support from my superiors. (17) I get enough appreciation from superiors. (18) I always have to make quick decisions. (19) I always have to look perfect. (20) (Two items): The company demands “friendliness” from me towards the guests in every situation/The company demands a “permanent smile” from me towards the guests, *r* = 0.69. (21) I have irregular breaks. (22) In a turnaround, I have the opportunity to take a short break. (23) I have retreat facilities available to me on board. (24) I have irregular eating habits.

* *p* < 0.05

** *p* < 0.01

*** *p* < 0.001.

**Table 4:** Correlations of experiencing serious events and contact with time differences with DASS-scores, sample 1 May 2019.

S. No		No (0)	Yes (1)	Depression	Anxiety	Stress
1.	Severe turbulences	40	65	−0.004	0.178	0.205^*^
2.	Emergency landings	90	15	0.021	−0.060	−0.002
3.	Death on board	84	21	−0.182	−0.012	−0.038
4.	Contact with time zone differences	41	64	0.059	0.074	0.160

N = 105. (1) I have already had an experience/s with a severe event/s in the form of severe turbulence. (2) I have already made an experience/s with a severe event/s in the form of an emergency landing. (3). I have already had an experience/s with a severe event/s in the form of death on board. (4) Do you come into contact with time zone differences?

* *p* < 0.05

** *p* < 0.01

*** *p* < 0.001.

“Fatigue”: I suffer from fatigue due to too little rest/I suffer from fatigue due to more than two sectors per day/I have sleeping problems when on early missions/I have sleeping problems when on late missions/I often suffer from jet lag fatigue due to time zone differences/I have problems changing time zones frequently within a month/I often have problems flying over time zones during a flight (*α* = 0.86). Respondents, who were not affected by time zone differences, had to answer only the first four fatigue items.

“On-call duty”: The duration of on-call duty (8–12 h a day) is too long/Waiting for calls from on-call duty is nerve-racking/Short-term calls from on-call duty are nerve-racking (*α* = 0.84).

“Duty roster design”: My days off are evenly distributed over the month/My duty roster is generally stable/My duty roster is generally employee-friendly/My roster wishes (days off and early/late assignments) are generally considered (*α* = 0.80).

### Statistical Procedures

Statistical evaluations were computed in SPSS 25 (IBM). The significance of the frequency comparisons was assessed by chi-square tests, and Odds Ratios (OR) were also calculated. Mean value comparisons were performed by t-tests for independent samples. Effect sizes are given as Cohen’s d. Correlation analyses were calculated as Pearson correlations (*r*). Dichotomous variables were dummy-coded. Cronbach’s *α* was calculated for the respective scales of the survey.

## Results

At the beginning of this study, we assumed that the particular stressors of flight attendants might correlate with an accumulation of psychological problems in this group of employees. We followed this assumption in a first survey in May 2019 (*N* = 105): the DASS-21 revealed group scores for depression of *M* = 3.56 (*SD* = 4.24), for anxiety of *M* = 1.96 (*SD* = 2.49), and for stress of *M* = 4.62 (*SD* = 3.73). Eight (8%) individuals showed clinically relevant symptoms of depression, six (6%) symptoms of anxiety, and eight (8%) symptoms of stress (with scores above the respective cut-offs). These numbers are actually the same or lower than those for the reference group of a (healthy) student sample (*N* = 413) of the DASS-21 [Nilges and Essau, 2015; depression: *M* = 4.1 (*SD* = 3.0), 8% above cut-off; anxiety: *M* = 2.5 (*SD* = 1.0), 12% above cut-off; stress: *M* = 5.9 (*SD* = 5.0), 13% above cut-off].

There are significant positive correlations of the symptoms of depression, anxiety, and stress with subjective stressors, namely, time pressure and work intensity, fatigue, the psychological and physical demands of the flight profile, and the burdens of on-call duty. Negative correlations were found with the working climate and the ability to cope well with physical conditions such as changing cabin pressure, air-conditioning, or noise (Table 3). The experience of monotony, underload, or lack of professional challenges correlates only with the symptoms of depression, whereas overload correlates with symptoms of anxiety and stress. The experience of support from colleagues and appreciation from the supervisor is negatively correlated with the stress symptoms; the experience of support from the supervisor is also negatively correlated with depression symptoms. Difficulties in communicating with passengers correlate positively and a favorable perception of the duty roster design correlate negatively with depression and stress. No correlations were found with the ability to make quick decisions, with the requirements for a perfect appearance, friendliness, or a permanent smile, with the regularity of breaks and retreats on board. Depression and stress show positive correlations with irregular eating habits and negative correlations with opportunities of taking a short break in a turnaround. There are no significant correlations with regard to severe events, apart from the positive correlation between severe turbulence and stress symptoms. For example, the experience of emergency landings or death on board has no connection with symptoms of depression, anxiety, or stress (Table 4).

Sample 2 (*N* = 1119) was acquired in April 2020 at the first peak of the corona crisis, when passenger numbers had declined by 99 and >90% of planes remained grounded. The majority of flying crew members (87%) of this sample were actually not flying anymore but benefited from the afore-mentioned short-work compensatory scheme. The DASS-21 revealed here group scores for depression of *M* = 6.49 (*SD* = 4.94), for anxiety of *M* = 2.72 (*SD* = 3.26), and for stress of *M* = 6.90 (*SD* = 4.97). Two hundred and fifty six (23%) individuals showed clinically relevant symptoms of depression, 161 (14%) of anxiety, and 266 (24%) of stress. These numbers are similar or higher than reported for the reference group of patients from psychotherapeutic ambulances for the dimensions depression and stress and lower for the dimension anxiety [Nilges and Essau, 2015; *N* = 145; depression: *M* = 6.3 (*SD* = 5.0), 19% above cut-off; anxiety: *M* = 4.1 (*SD* = 3.0), 20% above cut-off; and stress: *M* = 5.7 (*SD* = 5.0), 14% above cut-off].

In April 2020, 437 persons (39%) feared job losses as a result of the corona crisis (answer options summarized from “rather yes” to “yes” in absolute terms), whereas 385 persons (34%) did not fear this and 297 (27%) partly did. The fear of job loss is significantly related to the symptoms of depression, anxiety and stress (Table 2, e.g., *r* = 0.41 on depression symptoms). Before the corona crisis, only 32 (3%) of the respondents in sample 2 stated that they had existential fears (answers from “rather yes” to “yes,” summarized in absolute terms), whereas 393 (35%) of them stated that they had existential fears at the time of the second survey (during the corona crisis). This is a 12-fold increase. Depression symptoms correlated with the current existential fears to *r* = 0.43, with the existential fears before the corona crisis only to *r* = 0.08.

Eight hundred and eighteen (73%) of the respondents stated that their personal situation had worsened due to the crisis, 215 (19%) saw no change, and only 80 persons (7%) felt that their situation had improved. For the period after the crisis, 591 (53%) persons expected a deterioration, 153 (14%) no change, and 366 (33%) an improvement of their personal situation. There are negative correlations between a positively perceived change in the individual situation and all dimensions of the DASS-21. The most frequently given reasons for an improvement in their personal situation were: more time to rest, better sleep, more time for family, friends, pets, further professional training or reorientation, less stress, and healthier eating habits.

Sample 2 revealed interesting differences between employees who were still flying (*N* = 146) and those who had to stay home (*N* = 972). Employees who still flew showed significantly lower mean values in the areas of depression and stress, but with a lower effect strength. On the other hand, significantly more clinical anxiety symptoms were observed among colleagues who were still flying (Table 5).

**Table 5:** *T*-tests and Chi-square tests comparing DASS-scores of cabin crew members still flying with non-flying ones in April 2020.

	Still flying^a^		Non-flying^b^			
	*M*	*SD*	*M*	*SD*	*T*	*d*
Depression	5.66	4.95	6.61	4.93	−2.17^*^	0.19
Anxiety	2.86	3.67	2.70	3.20	0.50	0.05
Stress	6.12	4.91	7.01	4.97	−2.03^*^	0.18
**Clinically relevant symptoms**	**Incidence**	**%**	**Incidence**	**%**	**χ^2^**	**Odds Ratio^c^**
Depression	25/146	17.1	230/972	23.7	3.08	1.50
Anxiety	30/146	20.5	130/972	13.4	5.33^*^	0.60
Stress	27/146	18.5	238/972	24.5	2.52	1.43

a *N* = 146

b *N* = 972

c Comparison non-flying with still flying group

* *p* < 0.05.

A comparison between the samples before and during the COVID-19 pandemic showed a significant group difference as an increase in the categories anxiety, depression, and stress (Table 6). The effect sizes are *d* = 0.63 for depression symptoms, *d* = 0.26 for anxiety symptoms, and *d* = 0.52 for stress symptoms. The incidence of clinically noticeable symptoms increased significantly in a year-by-year comparison: it tripled for depression and stress (from 8 to 23% and 24%; odds ratios of 3.6 and 3.8, respectively) and more than doubled for anxiety (from 6 to 14% and odds ratio of 2.8).

**Table 6:** *T*-tests and Chi-square tests comparing DASS-scores between flying cabin crews sample May 2019 and April 2020.

	May 2019^a^		April 2020^b^			
	*M*	*SD*	*M*	*SD*	*T*	*d*
Depression	3.58	4.24	6.49	4.94	−6.62^***^	0.63
Anxiety	1.96	2.49	2.72	3.26	−2.90^**^	0.26
Stress	4.62	3.73	6.90	4.97	−5.79^***^	0.52
**Clinically relevant symptoms**	**Incidence**	**%**	**Incidence**	**%**	**χ**^2^	**Odds Ratio**^c^
Depression	8/105	7.6	256/1119	22.9	13.21^***^	3.60
Anxiety	6/105	5.7	161/1119	14.4	6.13^*^	2.77
Stress	8/105	7.6	266/1119	23.8	14.41^***^	3.78

a *N* = 105

b *N* = 1119

c Comparison April 2020 with May 2019

* *p* < 0.05

** *p* < 0.01

*** *p* < 0.001.

Characteristics of individuals in both samples are only very weakly related to depression, anxiety, and stress symptoms (Table 7). In sample 2, the age correlates negatively with the symptomatology, i.e., the younger respondents appear more prone to depression, anxiety, and stress. These effects are, however, small. Gender-specific effects are very small at most.

**Table 7:** Correlations between depression, anxiety and stress with characteristics of the persons.

	May 2019^a^			April 2020^b^		
	Depression	Anxiety	Stress	Depression	Anxiety	Stress
Gender (male = 0; female = 1)	0.020	0.014	0.047	0.006	0.041	0.066^*^
Position (flight attendant = 0; purser/ette = 1)	−0.035	0.068	0.113	−0.084^**^	−0.077^**^	−0.089^**^
Employment contract (fixed-term = 0; permanent = 1)	−0.098	0.067	0.136	−0.069^*^	−0.036	−0.066^*^
Age	−0.044	0.033	0.030	−0.159^**^	−0.119^**^	−0.130^**^
Number of children	−0.109	−0.071	−0.065	−0.024	0.004	0.057
Years of work experience	−0.127	−0.055	−0.040	0.029	0.041	0.008
Employment level	0.038	0.015	0.082	0.030	0.010	−0.040

a *N* = 105

b *N* = 1119

* *p* < 0.05

** *p* < 0.01

*** *p* < 0.001.

## Discussion

The results show that, as a consequence of the COVID-19 pandemic, the mental health status of flying cabin crews has significantly deteriorated, reaching an incidence of clinically conspicuous persons that is otherwise found only in the group of outpatients in psychotherapeutic clinics (Nilges and Essau, 2015). Persons who continued to fly during the crisis showed even more clinically relevant anxiety symptoms. Fear of infection with SARS-CoV2 would be a plausible explanation. For this fear, the terms coronaphobia (Asmundson and Taylor, 2020) and COVID-19 anxiety (Elhai et al., 2020) have been coined. For employees from various types of industries in Japan, a positive association has been found between the number of anti-COVID-19 measures taken and the fears and worries about the disease, which may reflect an increased awareness (Sasaki et al., 2020).

The comparison of the samples from May 2019 to April 2020 impressively shows the protective character of work. The frequencies of clinical abnormalities in sample 1 are comparable to the healthy reference sample (Nilges and Essau, 2015), despite the fact that the regular job of flight attendants comprises a whole range of potentially very stressful elements. The overall low level of depression, anxiety, and stress in sample 1 can also be explained by good personnel selection, which includes, e.g., stress resistance as a selection criterion (Herpertz et al., 2016). It is possible that self-selection also plays a role, during job application and by deciding whether to stay in the job or not.

Younger people in sample 2 were even more affected by symptoms of depression, anxiety, and stress than older ones. A first explanation for this could be that younger persons still have a longer professional future ahead of them and therefore consider the consequences of losing their job to be more serious. A second reason might be the general conditions for compulsory redundancies in Germany. Social compatibility is a high priority, which means that employees who have been with the company for a longer period of time are more likely to keep their jobs. However, no correlation between professional experience and symptoms was found.

Correlations of gender and hierarchical positions with the symptoms of depression, anxiety, and stress did not become obvious in sample 1. However, due to the larger sample size, they were rather significant in sample 2: Female persons show slightly more stress symptoms than their male colleagues, and ordinary flight attendants have higher values in all three dimensions than pursers/ettes. Subjectively experienced job burdens, fear of the future, and lack of prospects correlate all with depression, anxiety, and stress symptoms.

The found effect strengths of the mean values in our year-on-year comparison are comparable to those from meta-analyses of unemployment. Unemployment leads to a reduction in the well-being (*d* = 0.38; McKee-Ryan et al., 2005) and impairment of mental health (*d* = 0.51; Paul and Moser, 2009), with little habituation (Clark et al., 2008) and leads to long-term general health impairments (Daly and Delaney, 2013). In the worst case, unemployment can trigger suicide (Milner et al., 2014). Jahoda and her colleagues were already able to show the destructive course of unemployment in 1933 (Jahoda and Paul, 1933). The current crisis impressively demonstrates, here by the example of flight attendants, what a destructive effect already the threat of unemployment can have.

The protective factors of work are taken into account, for example, in Jahoda’s model of latent deprivation (Jahoda, 1981). According to this model, work has five significant psychological functions: it structures time, conveys status, creates meaning, enables social contacts, and integration into social goals. The loss of these latent functions of work through unemployment can damage mental health. The results of sample 1 not only show the protective character of work activity, but also demonstrate that it compensates for many work-related stressors. Starting points for occupational health and safety can be, for example, the enabling of breaks in turnaround and the support of regular eating habits. A further goal could be to sensitize supervisors even more to the support and appreciation of employees. This could have a positive effect on stress and depression symptoms.

The current crisis requires financial support for companies and individuals through state structures (Holtemöller et al., 2020). In addition, occupational health protection, the identification of career prospects, and convincing occupational safety concepts to prevent infection appear to be of the highest priority in order to counteract long-term psychological problems in the workforce and the population. During this crisis, healthcare systems prioritized the treatment of COVID-19 patients in an unprecedented way. This study suggests that healthcare systems must also prepare for a wave of mental illness and that society must now take massive prophylactic measures (see also Holmes et al., 2020).

## Limitations

This study was conducted online in German and posted on German-speaking websites. This implies a possible geographic and language bias as well as biases by personal preferences and web-using habits. The two surveys in 2019 and 2020 were also conducted as independent cohort studies. Their anonymous design precluded a direct comparison of pre-crisis and in-crisis parameters of the same individuals. However, this was compensated by a much larger crisis sample in 2020. Symptoms of depression, anxiety, and stress were assessed through self-reports and did not represent diagnoses.

## Conclusion

This study revealed a tremendous negative impact of the COVID-19 pandemic on the mental health of cabin crews. Job insecurity and fear of the future, as well as contact restrictions in general and not being allowed to work cumulated in a sharp increase in symptoms of depression, anxiety, and stress. Conversely, working on the job can be considered protective. The scale of the current crisis may require individual preventive mental health measures for groups of employees, such as cabin crews.

## Data Availability Statement

The raw data supporting the conclusions of this article will be made available by the authors, without undue reservation.

## Ethics Statement

The studies involving human participants were reviewed and approved by Ethics Committee of the Private University of Applied Science PFH Göttingen, Germany. The patients/participants provided their written informed consent to participate in this study.

## Author Contributions

YG and DS designed the study, conducted data collection, acquisition, and management, and drafted the work. YG performed data analyses and wrote the manuscript. Both authors critically revised the text and have approved the final article.

### Conflict of Interest

The authors declare that the research was conducted in the absence of any commercial or financial relationships that could be construed as a potential conflict of interest.

## Footnotes

^1^https://ufo-online.aero/de/

^2^https://www.aero.de/
